# Functional improvement of unicompartmental knee arthroplasty compared with total knee arthroplasty for subchondral insufficiency fracture of the knee

**DOI:** 10.1038/s41598-023-45748-2

**Published:** 2023-11-16

**Authors:** Dae Keun Suh, Jun-Gu Park, Jaejoong Kim, Dong Won Suh, Seung-Beom Han

**Affiliations:** 1 Department of Orthopaedic Surgery, Kyung Hee University Hospital, Seoul, 02447 South Korea; 2 Department of Orthopaedic Surgery, Anam Hospital, Korea University College of Medicine, 73, Goryeodae-Ro, Seongbuk-Gu, Seoul, 02841 South Korea; 3 Department of Orthopaedic Surgery, Barunsesang Hospital, Seongnam, 13497 South Korea

**Keywords:** Medical research, Bone, Skeleton

## Abstract

Subchondral insufficiency fracture of the knee (SIFK) causes acute knee pain in adults and often requires surgical management. Unicompartmental knee arthroplasty (UKA) and total knee arthroplasty (TKA) are the two most common surgical treatments for SIFK. While both UKA and TKA have their advantages, there is no consensus for SIFK localized on the medial compartment. We hypothesized that patients with SIFK treated with UKA would show superior patient-reported outcomes compared to those who underwent TKA. A total of 90 patients with SIFK located medially were included in the TKA (n = 45) and UKA (n = 45) groups. Size of SIFK lesions were measured on MR images. Patient reported outcomes in the form of the Western Ontario and McMaster Universities Osteoarthritis Index (WOMAC), Hospital Special Surgery (HSS) scores, and Knee Society Scores (KSS) were assessed preoperatively, postoperative 6, 12 months, and at the final follow-up. There were no differences in the size of the SIFK lesion between two groups. At 6 months, WOMAC score was better in the UKA group than the TKA group (*p* < .01). Both groups had a significant improvement in WOMAC, HSS, and KSS scores at the final follow-up compared to preoperative scores. The UKA group had better range of motion of the knee preoperatively and postoperatively than the TKA group (*p* < .01 and *p* < .01). UKA group showed a higher relative risk than the TKA group in terms of complications (RR = 3.0) but with no statistical significance (*P* = 0.31). Unicompartmental arthroplasty and total joint arthroplasty can produce successful outcomes in patients with SIFK with proper patient selection, regardless of the size of SIFK lesion.

## Introduction

Subchondral insufficiency fracture of the knee (SIFK) results from repeated physiological stress on the knee joint and commonly involve the medial femoral condyle of the knee^1^. SIFK used to be commonly misnamed as spontaneous osteonecrosis of the knee (SONK) since it was first described by Ahlback et al. in 1968^2^. In 2000, Yamamoto and Bullough described that these lesions histologically represent subchondral insufficiency fractures, occurring more often in osteoporotic bone^3^. Recent studies have confirmed that mechanical overloading of the subchondral bone leads to subchondral insufficiency fractures of the knee^4,5^. Patients typically present with an acute onset of knee pain that worsens with time. SIFK is more common in women and is associated with meniscal tears in up to 80% of patients^6^. Studies have shown 3.4% and 9.4% incidence of SIFK in individuals older than 50 and 65 years of age, respectively^7^.

Various surgical options exist for SIFK. These include core decompression, high tibial osteotomy, and joint replacement surgeries. Patients in early stages can be treated with teriparatide, percutaneous drilling but have shown unsatisfactory outcomes in Knee Society Score (KSS)^8^. Patients with failed conservative treatment or patients with more advanced stages of the disease often receive joint replacement surgeries such as unicompartmental knee arthroplasty (UKA) or total knee arthroplasty (TKA). Generally, UKA provides several advantages over TKA^9,10^. These include the use of a smaller operation port, preservation of the uninvolved compartment, decreased bone loss, physiological reconstruction of the native knee, and a quick postoperative recovery^11,12^. However, UKA has also been associated with bone collapse owing to a weak supporting bone and loosening of the tibial component^13^. Aseptic tibial loosening has been cited as the most common cause of early failure in UKA^14,15^. TKA has advantages in terms of the stability and longevity of the implant, thus the worry of a weak supporting bone is less of a concern. TKA requires sacrificing the lateral compartment, causing damage to the possibly unaffected lateral compartment^16^. Adequate patient selection and adherence to surgical indications for UKA and TKA are key factors for success. To our knowledge, few studies have addressed and compared the outcomes of UKA and TKA for SIFK^12,16,17^.

The purpose of this study was to compare the outcomes of UKA to those of TKA for patients with SIFK on the medial femoral condyle. Considering the advantages of UKA, we hypothesized that patients with SIFK treated with UKA would show superior patient reported outcomes and less postoperative complications compared to those who underwent TKA.

## Materials and methods

We retrospectively searched our institution’s prospectively collected database after receiving approval from the Institutional Review Board of Korea University Anam Hospital (2022AN0348). All research conducted was performed in accordance with the relevant guidelines and regulations.. The inclusion criteria were patients who underwent either TKA or UKA for SIFK confirmed by magnetic resonance imaging (MRI) between January 2007 and December 2020. All radiographs and MRI scans were reviewed by two surgeons (S.D.K. and H.S.B.). The exclusion criteria were patients with SIFK in the lateral compartment, patients with a follow-up of less than two years, and patients with incomplete medical records. Surgery was performed by a single senior surgeon (H.S.B.) with more than 200 TKA and 50 UKA cases per year.

**Figure 1:**
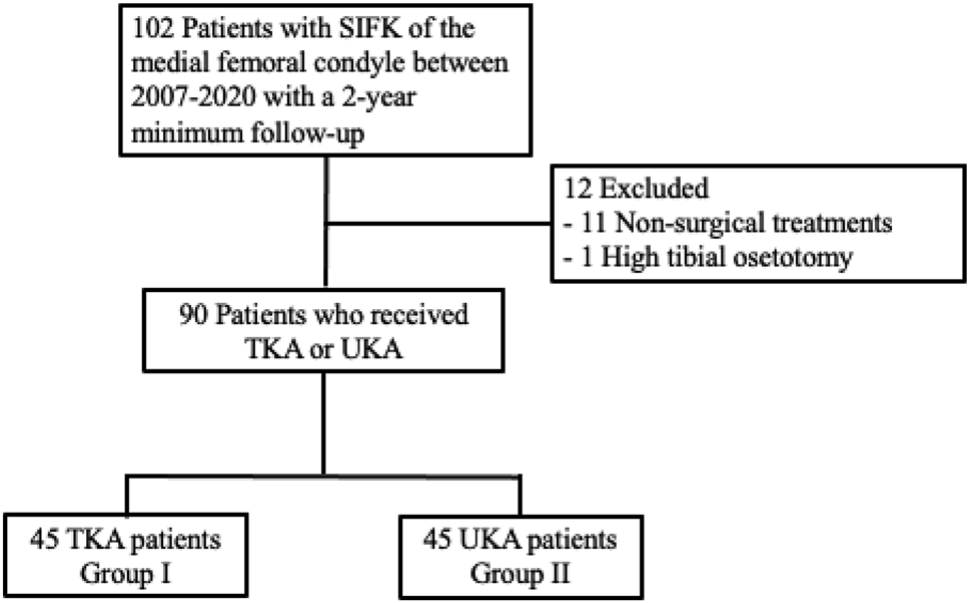
Flowchart illustrating the selection of patients.

UKAs met the standard applicability criteria, including minimal arthritic change of the lateral or patellofemoral compartment, a stable knee joint, less than 15° of flexion contracture, and a manageable limb alignment^18^. Depending on the type of surgery received, the cohort was divided into two groups: TKA and UKA. Of the eligible 102 patients, 11 patients did not receive surgical intervention and one patient received high tibial osteotomy. A total of 90 patients were included in the study, of which 45 patients received TKA and 45 patients received UKA. (Fig. 1).

For the TKA group, a cemented posterior cruciate-substituting design (e.motion Total Knee System, B. Braun Melsungen AG) was used with patellar resurfacing. For the UKA group, a cemented implant with a mobile-bearing design (Zimmer Biomet, Warsaw, Indiana, USA) was used. Patients in both groups were subjected to the same postoperative protocol. On the second postoperative day, the hemo-vac was removed, and range of motion (ROM) exercises were started. Patients would then progress to tolerable weight-bearing.

**Figure 2:**
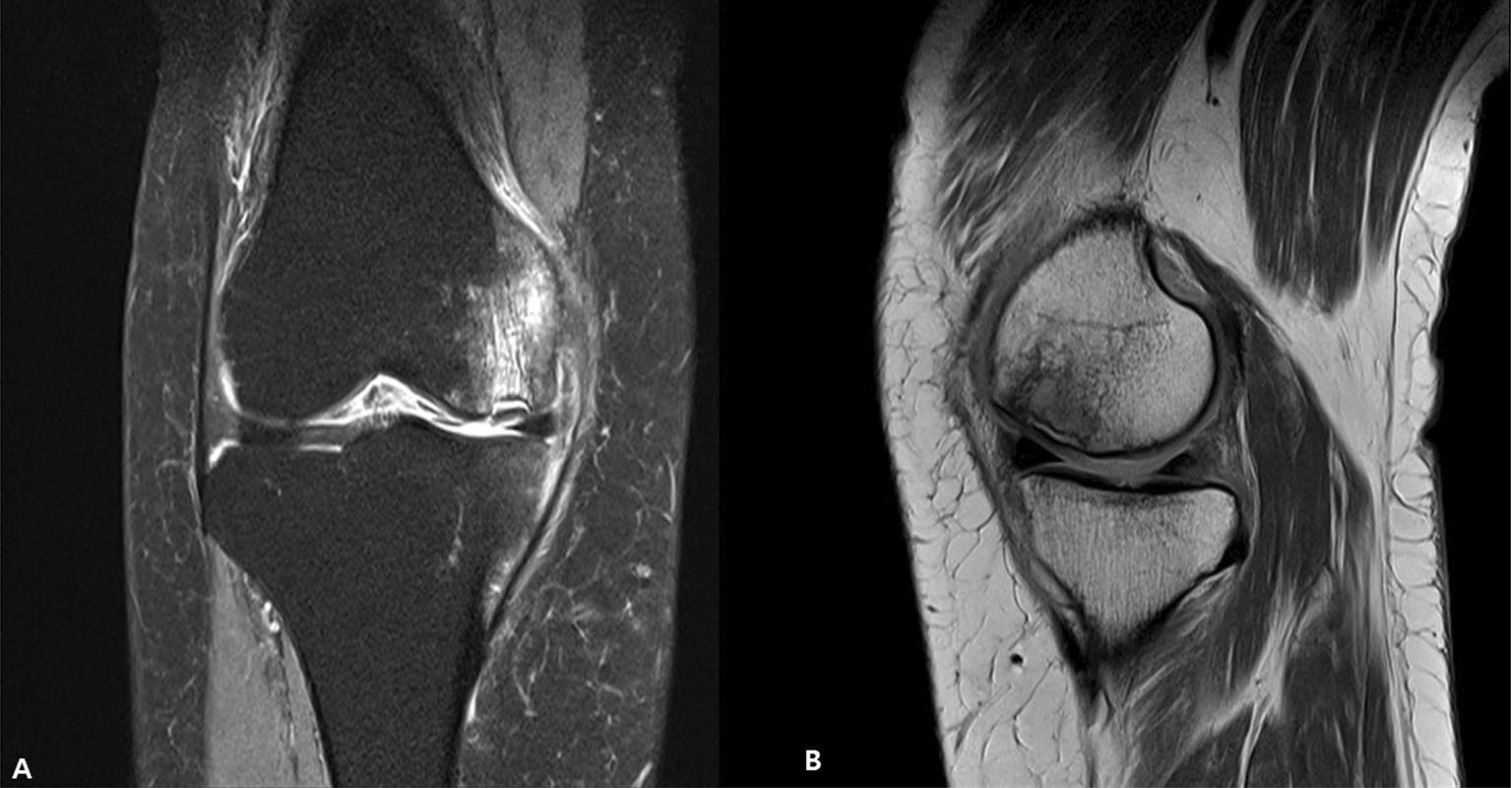
Coronal T2-weighted MRI of SIFK (left). Sagittal T1-weighted MRI (right). MRI, magnetic resonance image. SIFK, subchondral insufficiency fracture of the knee.

**Figure 3:**
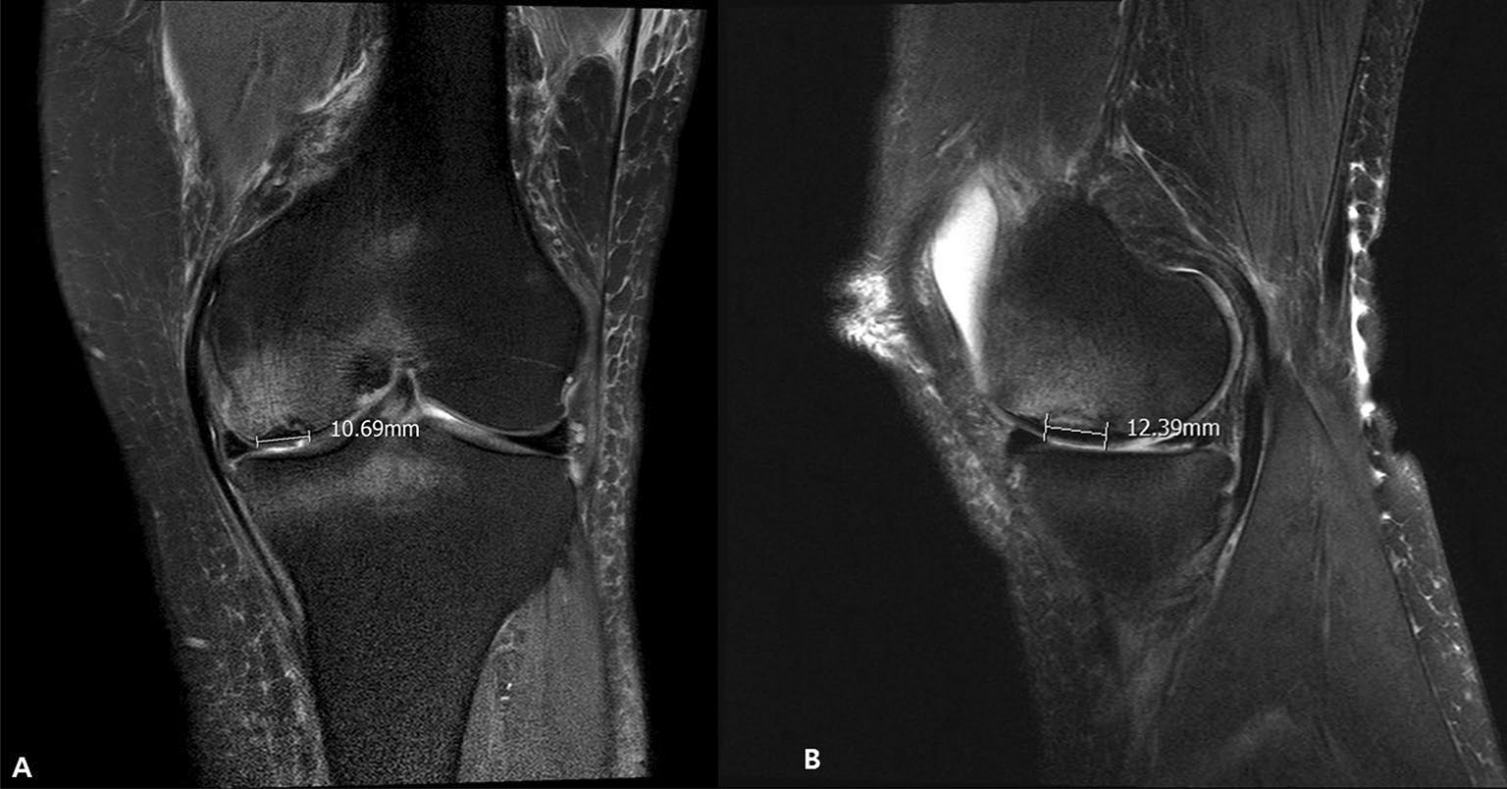
Coronal measurement of the SIFK lesion (left). Sagittal measurement (right). SIFK, subchondral insufficiency fracture of the knee.

All patients received preoperative MRI scans. MRI characteristics of SIKF include a subchondral area of low signal intensity on T2-weighted images, focal epiphyseal contour depressions, and lines of low signal intensity in the affected condyle^19^. For the present study, a SIFK lesion was defined as a low signal intensity focus beneath the articular surface in the weight-bearing area of the tibiofemoral compartment on T1-weighted images and a central focal linear subchondral low signal intensity or focal subchondral bone plate impaction with marked surrounding bone marrow edema on fat-suppressed T2-weighted images^20^ (Fig. 2). The lesion size was measured on coronal and sagittal MR images, and the largest lesion was measured in length (sagittal), width (coronal), depth (deepest portion measured in either coronal or sagittal view)^21^ (Fig. 3).

**Figure 4:**
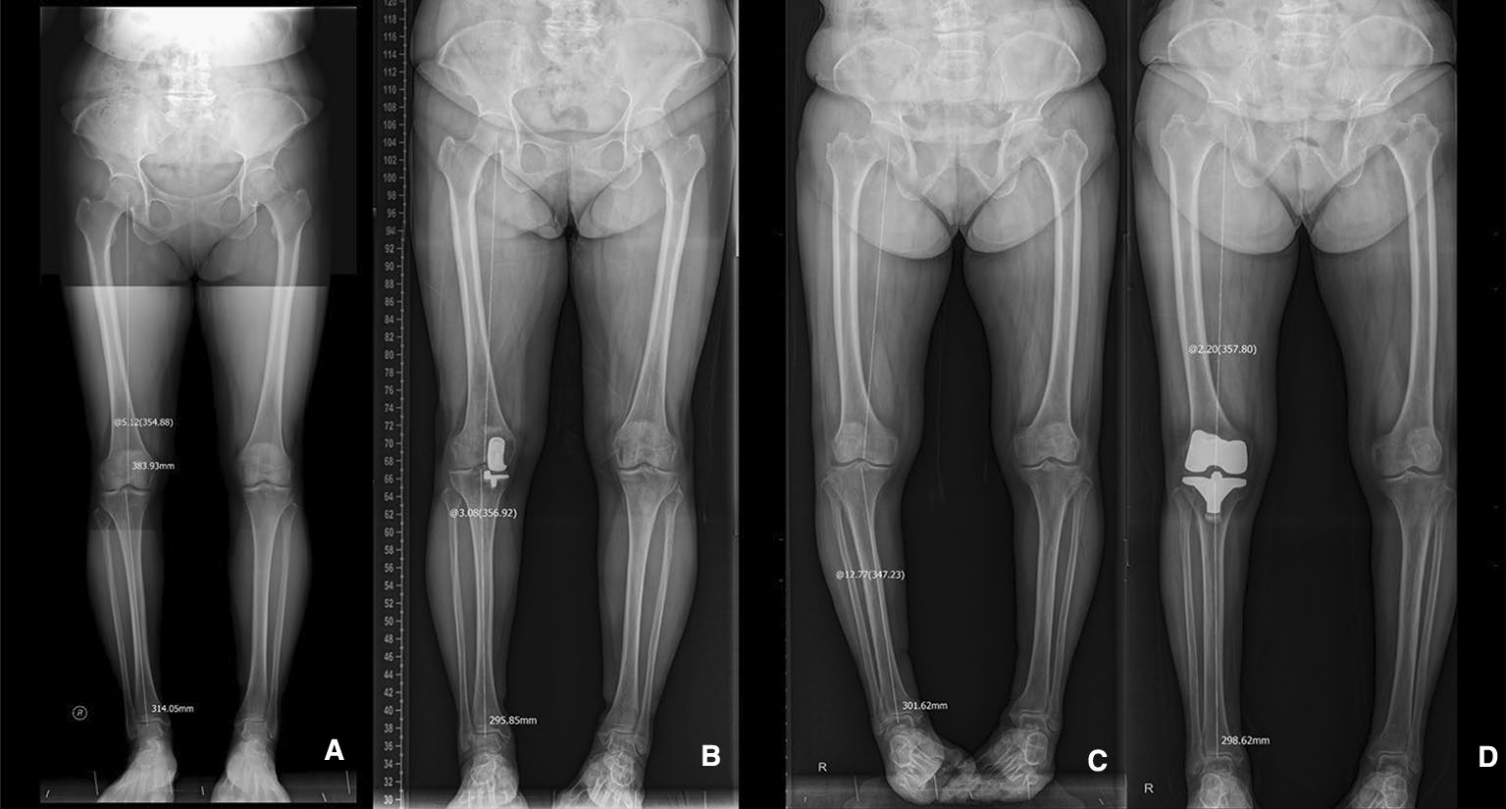
HKA angles for UKA and TKA. Preoperative and postoperative HKA angle for UKA (**A**,**B**). Preoperative and postoperative HKA angle for TKA (**C**,**D**). HKA, hip-knee-ankle. UKA, unicompartmental knee arthroplasty. TKA, total knee arthroplasty.

Anterior–posterior (AP), lateral, and full-length standing AP radiographs were obtained preoperatively and at the final follow-up. The preoperative and postoperative hip-knee-ankle (HKA) angle, defined as the angle between the femoral and tibial mechanical axes, were measured^22^ (Fig. 4).

Patient reported outcomes in the form of the Western Ontario and McMaster Universities Osteoarthritis Index (WOMAC), Hospital for Special Surgery (HSS), and Knee Society scores (knee score (KSKS) and function scores (KSFS)) were assessed preoperatively, postoperative 6 months, 1 year, and at the final follow-up^23,24^. Patient satisfaction scores from a patient-reported survey with scores ranging from 0 to 5, with 0 being the worst score and 5 being the best score (extremely satisfied), were obtained at the final follow-up. ROM was measured by a single surgeon (J.G.P.) preoperatively and at the final follow-up.

Continuous variables were presented as mean ± standard deviation and categorical variables as numbers and percentages. Normal distribution of the data was tested using Kolmogorov–Smirnov test. Demographic characteristics and radiological and clinical data of the patients in the two groups were compared using two-tailed Mann–Whitney test and Student’s *t-*test, as appropriate. Differences between categorical variables were evaluated using chi-square test. A post hoc power analysis was performed, and our available sample size required 51 patients per group to achieve an 80% power to detect a significant change. Relative risk was calculated by dividing the complication rate in the UKA group by the risk of complication from the TKA group. Statistical significance level was set at 0.05. All data were analyzed using Statistical Package for the Social Sciences version 20.0 software (IBM Corp., Armonk, NY, USA).

## Results

A total of 45 and 45 patients with SIFK were included in the TKA and UKA groups, respectively. There were no differences in sex ratio, age, BMI, and follow-up between two groups (Table 1). UKA group had a significantly shorter operation time (67 vs 79 min; *p* < 0.01) but with no difference in hospital stay (7.2 vs. 7.6 days; *p* = 0.22). The two groups differed in two preoperative parameters—preoperative ROM and HKA angle (Table 1). UKA cases showed better preoperative and postoperative ROM (*P* < 0.01 and *P* < 0.01). The mean preoperative HKA angle of the TKA group was 8.76 ± 3.1 degrees varus and that of the UKA group was 1.06 ± 3.43 degrees varus.

**Table 1:** Preoperative demographic, radiographic, and clinical data.

Variables	TKA group (n = 45)	UKA group (n = 45)	*P*-value
Sex, F:M	40:5	41:4	0.82
Age, y	70.9 ± 7.6	72.7 ± 6.6	0.60
BMI, kg/m^2^	25.9 ± 3.3	25.5 ± 1.8	0.60
Follow-up, m (range)	57.4 ± 34.9 (24–124)	45.0 ± 18.8 (24–91)	0.12
Pre-HKA angle, °	8.76 ± 3.1 varus	1.06 ± 3.43 varus	0.02
Pre-ROM, °	126.1 ± 12.7	137.9 ± 4.6	< .01

*Data are presented as mean ± standard deviation unless otherwise indicated. BMI, body mass index. HKA, hip-knee-ankle. ROM, range of motion.

No differences in SIFK lesions were found between the TKA and UKA groups. In the TKA group, the average size of lesion was 11.32 mm in width, 17.65 mm in length, and 4.81 mm in depth. The average size of lesion in the UKA group was 13.79 mm in width, 19.62 mm in length, and 5.73 mm in depth (Table 2).

**Table 2:** Size of SIFK lesions.

Size of lesion (MFC)	TKA group (n = 45)	UKA group (n = 45)	*P*-value
Coronal	11.32 ± 4.66 mm	13.79 ± 2.50 mm	0.09
Sagittal	17.65 ± 7.36 mm	19.61 ± 5.88 mm	0.33
Depth	4.81 ± 1.74 mm	5.72 ± 2.50 mm	0.09

*Data are presented as mean ± standard deviation unless otherwise indicated. SIFK, subchondral insufficiency fracture of the knee. MFC, medial femoral condyle.

Patient-reported outcomes of the UKA group showed better WOMAC and KSFS scores than the TKA group at postoperative 6 months, although the final follow-up the patient-reported outcomes were not significantly different between the groups (Table 3). UKA group showed a higher satisfaction score of 4.4 than the TKA group’s 4.0 but with no significance (*P* = 0.08) (Table 4).

**Table 3:** Patient-reported outcomes at each time point.

	TKA group (n = 45)	UKA group (n = 45)		*P*-value
KSKS				Time < 0.01; Group 0.78; Time · Group 0.19
Preoperative	52.5 ± 11.0	49.5 ± 14.2		0.367
6 month	89.3 ± 7.7	92.8 ± 4.5		0.055
12 month	93.7 ± 5.9	94.9 ± 4.9		0.441
Last follow-up	94.1 ± 6.5	93.9 ± 6.9		0.899
KSFS				Time < 0.01; Group 0.07; Time · Group 0.37
Preoperative	44.6 ± 10.9	45.4 ± 9.5		0.76
**6 month**	**69.3 ± 13.8**	**81.7 ± 11.8**		** < .01**
12 month	76.0 ± 13.9	82.2 ± 12.3		0.09
Last follow-up	81.9 ± 13.5	80.4 ± 13.1		0.69
HSS score				Time < .01; Group 0.75; Time · Group 0.68
Preoperative	60.2 ± 7.7	58.4 ± 8.5		0.39
6 month	86.8 ± 7.0	89.7 ± 5.0		0.09
12 month	91.2 ± 5.0	91.3 ± 5.3		0.98
Last follow-up	91.9 ± 5.8	92.1 ± 5.0		0.89
WOMAC score				Time < .01; Group 0.11; Time · Group 0.96
Preoperative	61.3 ± 13.8	61.1 ± 12.4		0.94
**6 month**	**20.2 ± 8.4**	**14.4 ± 6.5**		** < .01**
12 month	13.8 ± 9.4	10.6 ± 6.6		0.16
Last follow-up	9.5 ± 11.0	8.6 ± 8.2		0.73

*Data are presented as mean ± standard deviation unless otherwise indicated. KSKS Knee Society Knee Score. KSFS Knee Society Function Score. HSS Hospital for Special Surgery. WOMAC Western Ontario and McMaster Universities Arthritis Index.

**Table 4:** Comparions of clinical outcomes and satisfaction scores at the final follow-up.

	TKA group (n = 45)	UKA group (n = 45)	*P*-value
Post-HKA angle, °	3.21 ± 2.3 varus	0.48 ± 2.21 valgus	< .01
Post-ROM, °	130.9 ± 9.0	139.5 ± 2.2	< .01
Satisfaction score	4.1 ± 0.9	4.4 ± 0.5	0.08
Complications	1	3	0.31

*Data are presented as mean ± standard deviation unless otherwise indicated. HKA, hip-knee-ankle. ROM, range of motion.

There was one complication in the TKA group where the patient was admitted for 5 more additional hospital days for an elevated creatinine level after surgery. The patient recovered without further complications. There no revision cases in the TKA group during the follow-up period. There were three complications (6.7%) in the UKA group. All three cases were due to dislocation or breakage of the mobile bearing. Two of these patients developed bearing dislocation within 6 months of receiving the operation and one patient developed bearing breakage after 9 years of the surgery. All three patients received bearing exchange with a thicker bearing. There were no patients in the UKA group who needed conversion to TKA after a mean follow-up of 45.0 ± 18.8 months (range 24–91 months). There were no significant differences between the two groups (Table 4) but based on the complication rates, UKA showed a higher relative risk of 3.0 compared to TKA.

Patients in the TKA group showed a significant change in alignment from 8.76 ± 3.1 varus to 3.21 ± 2.3 varus (*P* < 0.01). Patients in the UKA group showed a change in alignment from 1.06 ± 3.43 varus to 0.48 ± 2.21 valgus but with no statistical significance (*P* = 0.21) (Table 4).

## Discussion

The most important findings of this study are that the UKA group showed better functional scores than the TKA group at postoperative 6 months and both groups showed equally successful patient reported outcomes for patients with SIFK of the medial compartment.

Favorable results of UKA have been consistently reported in literature for patients with knee osteoarthritis and SIFK^25–30^. However, the literature comparing the effects of TKA and UKA in patients with SIFK is limited. The findings of this study are notable because, to our knowledge, only two previous studies have directly compared the results of UKA and TKA for SIFK^16,17^. The current study compared the results of a cohort of 90 patients from medial SIFK treated with UKA or TKA with an average follow up of 4 years. UKA showed more improved KSFS and WOMAC scores than TKA at postoperative 6 months regardless of the size of the SIFK lesion. In a clinical setting, improved function and lower pain allows patients to be able to participate in physical therapy or rehabilitation with more ease^31^. Patients in the UKA group showed a higher complication rate than the TKA group with a relative risk of 3.0 but with no significance (*P* = 0.31).

In 2005, Radke et al*.* performed a retrospective analysis on 23 UKA and 16 TKA patients with SIFK. They showed that TKA has better long-term clinical results than UKA due to better implant fixation in necrotic lesions. They also reported higher revision rates with lower clinical outcomes in UKA patients from the development of secondary arthritic changes and potential osteonecrosis transformations of other knee compartments^16^. More recently, Flury et al*.* retrospectively evaluated 27 UKA and 34 TKA patients with SIFK after a mean follow-up of 6.6 years. They found that UKA showed slightly better functional outcomes but with a higher complication rate. They concluded that UKA is a valid treatment option for SIFK, producing good functional results and low failure rate which are comparable to those of UKA for osteoarthritis, and that size of lesions do not seem to influence the results^17^.

Patient selection is critical when making the decision to treat patients with either UKA or TKA. In a systematic review by Myers et al*.*, the outcomes of 148 TKA knees and 64 UKA knees with SIFK were compared. The global knee scores after TKA for spontaneous osteonecrosis were better than those after UKA for spontaneous osteonecrosis, but the authors questioned the adequacy of patients selected for UKA^12^. Despite the differences in some of the demographics between the UKA and TKA group, the present study only included patients with SIFK on the medial compartment. From our knowledge, only one study previously compared the results of unicondylar and total knee arthroplasty with SIFK specified to a single compartment^16^. The patient selection of UKA in the study by Radke et al*.* can be questioned as the authors attributed worse clinical outcomes and higher revision rate in UKA mostly to secondary osteoarthritis changes in the other compartments ^16^. The results of the present study showed that UKA showed similar long term clinical results with no loosening of implants. Similarly, several meta-analyses have shown that UKA can produce similar survival rates and clinical outcomes for SIFK and medial osteoarthritis with a proper selection of patients ^32,33^.

The widely accepted pathophysiology of SIFK is an insufficiency fracture of the subchondral bone, which commonly affects older women with a low BMI^3^. The most common location of SIFK is the medial femoral condyle. Studies have found that medial meniscal tears are also prevalent in patients with SIFK^34^. Co-involvement or sole involvement of the lateral compartment is an indication to avoid UKA and to perform TKA. As SIFK lesions are commonly found in the weight-bearing portions of the knee, subchondral bone quality has always been a concern when performing UKA or TKA. Large epiphyseal lesions are associated with worse prognosis than small and medium lesions, a phenomenon observed in the hip^8^. To successfully treat SIFK with arthroplasty, Guo et al. emphasized the importance of recognizing the presence of bone defects, scooping out the avascular portion, and filling lesions with autologous bone grafts for lesions sized > 5 mm^35^. The current study showed that implant fixation is not a concern for UKA when treating patients with SIFK. Patients in both groups showed no implant loosening and without the use of autologous bone grafts or the need to scoop SIFK lesions. The location and size of the lesion did not affect the choice of surgery and thus did not affect patient reported outcomes. Size of lesions should not discourage surgeons from performing UKA if all parameters indicate that the patient is an adequate candidate. Although both UKA and TKA implants showed no signs of implant loosening, UKA group showed three cases with bearing malfunction. Associating bearing malfunction with SIFK needs to be investigated, but bearing dislocation and breakage is a specific complication seen in UKA implants with mobile bearing^36,37^. A systematic review and meta-analysis by Ro et al. showed that Asian patients were more likely to undergo reoperation for bearing dislocation than Western patients^38^. Bearing dislocation could be avoided by using a fixed bearing unicompartmental implant or a total knee implant.

There were several limitations to this study. First is that this is a retrospective study design with no randomization. Morever, a possible selection bias may exist due to the difference in some of the preoperative parameters between two groups, such as ROM and HKA angle. Range of motion was significantly improved in both groups, but varus alignment was more significantly improved in the TKA group. However, two groups showed no difference in lesion size, and the same postoperative protocols were assigned to both groups (Table 2). Recent randomized studies on the treatment of osteoarthritis showed that UKA and TKA showed no difference in early postoperative periods in terms of patient-reported outcomes and return to work and showed that UKA can shorten hospital stay while producing greater range of motion^39,40^. Small size of patients is another limitation. As the power analysis showed that each group required 51 patients, the sample size of this study did not meet the size required for the appropriate power analysis, but this mainly due to the rare nature of the disease. Despite the study being underpowered and the two groups having some differences, we believe this study provides valuable information for surgeons dealing with patients with SIFK. This is one of the largest cohort of patients that compare the UKA and TKA for SIFK with a mean follow-up of 4 years.

Despite some key limitations mentioned above, this study provides clinical insight on the treatment of SIFK, showing that UKA is a reliable surgical modality of choice regardless of size of lesions in the medial compartment. UKA and TKA show equally successful patient reported outcomes, while UKA showing better functional and pain scores (KSFS and WOMAC) at 6 months postoperatively. Further investigation involving randomized controlled prospective study is needed to compare the outcome of the surgical modalities more accurately.

## Conclusion

In summary, regardless of the size of the SIFK lesion, UKA can show faster recovery and produce equally satisfying outcomes as TKA with proper patient selection. For SIFK lesions confined to MFC, the patient’s preoperative radiological status and characteristics as well as the surgeon’s expertise should be considered when selecting the surgery of choice.

## Data Availability

The datasets generated during and/or analysed during the current study are not publicly available due to the protection of hospital and patient data but are available from the corresponding author on reasonable request.
